# The association between boredom proneness, functional status, and views on ageing in geriatric patients

**DOI:** 10.3389/fpsyg.2025.1657437

**Published:** 2025-11-06

**Authors:** Anna Lena Küstner, Aline Schönenberg, Tino Prell

**Affiliations:** Department of Geriatrics, Halle University Hospital, Halle, Germany

**Keywords:** boredom, older adults, views on aging, geriatric, functional health, aging perception

## Abstract

**Background:**

Boredom proneness in later life has been linked to poorer psychological and functional outcomes, yet little is known about how individual Views on Ageing (VoA) influence boredom or whether boredom per se predicts rehabilitation success. We therefore examined (1) the cross-sectional associations of positive and negative VoA with boredom proneness, and (2) the longitudinal effect of boredom on functional gains during a two-week geriatric rehabilitation.

**Methods:**

In a sample of 120 inpatients (mean age 83.4 ± 6.4 years; 70.8% female) undergoing geriatric early complex rehabilitation, boredom was measured at admission using the eight-item Short Boredom Proneness Scale (SBPS). VoA were assessed with a 16-item questionnaire covering the domains physical decline, continuous growth, self-knowledge and social losses. Functional status was quantified by the Barthel Index at admission and discharge. We first fitted linear regression models of SBPS on VoA, adjusting sequentially for age, sex, living situation, education, and depressive symptoms (GDS). Next, we applied a longitudinal mixed-effects model to test SBPS as a predictor of functional improvement.

**Results:**

Negative VoA strongly predicted higher SBPS (*B* = 0.39, *p* < 0.001) even after full adjustment (adj *R*^2^ = 0.39). Higher SBPS was associated with smaller changes in functional status in unadjusted (*β* = −0.93, *p* = 0.014), partially adjusted (*β* = −0.77, *p* = 0.009), and fully adjusted models (*β* = −0.84, *p* = 0.012). ANCOVA confirmed a negative SBPS effect on discharge Barthel (*β* = −0.90, *p* = 0.002). Mixed modeling revealed a significant Time × SBPS interaction (*β* = −0.94, *p* = 0.010), indicating that each SBPS point reduced expected rehabilitation gain by 0.94 Barthel points (ICC = 0.19).

**Conclusion:**

Older patients holding negative VoA are more prone to boredom. This proneness is further associated with less functional recovery during rehabilitation. Interventions targeting maladaptive VoA and boredom may enhance engagement and improve rehabilitative outcomes.

## 1 Introduction

The progressive ageing of the population is a key issue for society, particularly in countries where the number of older people is steadily increasing (Bujard, 2022). This demographic shift opened up the discussion on healthy aging, now recognized as multidimensional with the inclusion of physical and cognitive health but also psychological well-being and social connectedness (Rowe and Kahn, 1997; Menassa et al., 2023). Especially the upkeep of quality of life emerges as a primary goal in scientific research and healthcare of older adults in the face of declining health, shifting the focus from restitution to well-being (Guyatt et al., 2007; Dinglas et al., 2018). While physical and biological aspects of aging have long-since been considered, recently, psychosocial aspects of aging have come into the focus of scientific research. This focus is motivated by a differentiation between biological age and subjective age. While biological age is informed by biochemical processes in the body, subjective age reflects how old a person feels based on a multitude of factors such as physical health, mental health, social comparison and societal norms (Diehl et al., 2014). While biological and subjective age mutually inform each other (Kwak et al., 2018), they are not identical (Stephan et al., 2015) and have even been shown to independently affect COVID-19 infection risk (Berezina and Rybtsov, 2021). While subjective age encompasses measures of physical health and functional status, it is further informed by psychosocial factors, expectations, and cognitive appraisals (Bergland et al., 2014; Hwang and Hong, 2019; Heimrich et al., 2022). Younger subjective age has been shown as beneficial for improved health outcomes (Westerhof et al., 2014; Wurm et al., 2017; Aftab et al., 2022; Westerhof et al., 2023) as it may dictate a person’s behavior (Montepare, 2020).

One of the complex and highly relevant cognitions associated with subjective age are attitudes towards aging and aging expectations (Abud et al., 2022; Wahl et al., 2022; Menassa et al., 2023). In research, so-called views of ageing (VoA), i.e., collective and individual perceptions of old age and ageing, are of central importance. They form as early as childhood and develop throughout the lifespan, as the process of aging is not solely restricted to advanced age but rather happens each day of life as a continuous process (Mendonça et al., 2018; Wareham, 2018; Whatley and Castel, 2020). These VoA are often ambivalent, ranging from negative stereotypes of physical frailty, loneliness and dependence, to positive images that emphasize continuous growth, independence and active participation in society (Altenberichtskommission, M.D.S, 2010). Separate from biological age, VoA reflect the idea that ageing is also a social construct depending on social comparison and the perception of one’s own physical and psycho-emotional development over time (Diehl et al., 2014; Overall, 2018; Diehl and Wahl, 2020). VoA have far-reaching implications for older people’s self-perceptions and influence not only their psychological well-being but also the way they organize their lives in old age (Kornadt et al., 2020). While VoA serve as an umbrella term covering a large span of implicit and explicit cognitions about the own aging experience as well as societal expectations (Klusmann and Kornadt, 2020; Wurm et al., 2025), they are often categorized into gain- or loss-related VoA, also labelled positive and negative VoA (Wurm et al., 2025). Positive VoA encompass the mindset that ageing, despite health-related challenges and decline, is still accompanied by lifelong learning and by the ability to realize plans and actively participate in life. They reflect the idea that older age does not hinder growth and improvement but instead brings forth wisdom and knowledge, helping older people navigate their wished and needs with more serenity(Wurm et al., 2007; Diehl et al., 2014; Wurm et al., 2025). VoA, via motivational pathways and expectations, may influence behavior (Lang and Rupprecht, 2019; Montepare, 2020). (Kornadt et al., 2020)Therefore, positive VoA are associated with active and healthy ageing, encouraging older adults to adopt health-promoting behaviors such as social interaction and physical activity, overall contributing to quality of life and life expectancy (Levy et al., 2002; Westerhof et al., 2023). In contrast, negative VoA reflect the perception that older age is characterized by physical decline, lack of resilience and capacity, as well as a social component that includes loss of social roles, respect, and isolation. Thus, negative VoA can lead to resignation, social isolation and a decline in quality of life. They can also increase susceptibility to mental health issues such as depression and loneliness (Low et al., 2013).

Boredom is a subjective emotional state that occurs when an activity or situation lacks meaning or appeal, leading to feelings of restlessness and irritation. It signals a desire for something more engaging or interesting, emphasizing the lack of meaning (Barbalet, 1999). Boredom can be considered both a trait, encompassing a multitude of situations and signaling a general tendency to experience boredom, as well as a state arising in certain situations (Harris, 2000; Hadjioannou, 2019). As a state experience, boredom serves as a signal accompanied by the strong urge to re-direct attention and seek a different task or situation, making it an often overlooked but crucial element in the daily lives of people (Bieleke et al., 2022). While frequently dismissed as a trivial emotion, persistent boredom can have substantial negative impact on health and well-being. The overall tendency to experience boredom in many different situation signals general proneness to boredom. Trait boredom may be a concept generally related to personality and self-control (Wolff and Martarelli, 2020), however, it may also be rooted in a lack of ability or opportunity to change the present situation, e.g., due to health-related circumstances. As the present research paper addresses geriatric patients who may be limited in their daily activities, boredom is treated as a trait. In the literature, this continued boredom is linked to adverse mental health outcomes such as cognitive impairment, depression and increased social isolation (Farmer and Sundberg, 1986; Finkielsztein, 2022; An et al., 2023; Ndetei et al., 2023). Moreover, the emotional distress associated with boredom may exacerbate physical health risks, including elevated heart rates and deteriorating cardiovascular parameters (Merrifield and Danckert, 2014; Finkielsztein, 2022). Due to its impact on motivation and activity via cognitive appraisal and lack of meaning in activities, boredom may be linked with maladaptive behavior (Bieleke et al., 2022) such as increased sedentary behavior and lack of motivation to improve health outcomes (Van Tilburg and Igou, 2012; Wolff et al., 2021). Thus, it is of interest to understand how boredom is linked with functional health and independence in daily tasks, which are commonly reduced in geriatric patients. Functional health, such as the ability to perform daily activities like eating and showering independently, cannot sufficiently be predicted by common health-related factors such as cognition and physical health (Heimrich et al., 2025), suggesting that motivational factors may be at play. These findings highlight the multidimensional consequences of boredom on both psychological and somatic levels.

As VoA shape how older adults expect their life to unfold and which beliefs they hold about aging processes, it is probable that VoA and boredom are related. Since VoA dictate behavior and activity levels as well as cognitive appraisals of health events and changes in well-being, negative VoA may be closely linked to boredom proneness due. Endorsing negative VoA such as the perception that older age is accompanied by physical and cognitive decline, increased loneliness and less respect may be accompanied by higher boredom proneness. In contrast, positive VoA such as the belief in lifelong learning and the ability to realizing plans is likely connected to lower boredom levels. A previous study has approached this topic by showing that the ability to be fully engaged and immersed in tasks on a daily basis was linked with more positive VoA and affect (Finnigan et al., 2025). However, the extent to which perceptions of ageing influence the subjective experience of boredom in older adults remains poorly understood. While existing research has explored the impact of boredom on quality of life and mental health, there is a notable lack of empirical data investigating how positive and negative VoA shape the perception and intensity of boredom, particularly in a geriatric context. Geriatric patients are defined by a high level of functional dependence, comorbidities and vulnerability (Sanford et al., 2020), potentially making them particularly prone to boredom. Previous research has shown that positive VoA can buffer the effect of health events and exacerbations of health decline (Wurm et al., 2008), while health events in turn may increase both positive and negative VoA depending on their social and emotional context (Rupprecht et al., 2022). In line with Rowe and Kahn’s model of successful aging as multidimensional (Rowe and Kahn, 1997), these results show that VoA are not only shaped by current health (Benyamini and Burns, 2020), making the interplay of VoA and boredom particularly relevant in acutely ill geriatric patients. Thus, understanding these interactions is crucial, as both VoA and boredom have significant potential to influence health outcomes.

To address these gaps, this study aims to examine the role of positive and negative VoA as factors associated with the experience of boredom in older people. By understanding these relationships, the research seeks to contribute to the development of targeted interventions that promote active, meaningful engagement and improve the overall quality of life for older adults. Moreover, we aimed to investigate if prone to boredom influences functional outcome in geriatric patient receiving geriatric treatment.

## 2 Materials and methods

### 2.1 Study design

This cross-sectional observational study was carried out between November 2023 and June 2024 at the Centre for Geriatrics in the South of Saxony-Anhalt, Germany (Zentrum für Altersmedizin im südlichen Sachsen-Anhalt, ZASSA). We included geriatric patients who received early complex rehabilitation treatment specifically designed for older people hospitalised with acute illness or injury, classified under the Operations and Procedures Key (OPS) system 8–550. This treatment spans across 14 to 21 days (mean stay duration 15.52 ± 4.24 days) and is provided by a multidisciplinary team including geriatricians, nurses, physiotherapists, occupational therapists, speech therapists, social workers, psychologists and other specialists. Patients were excluded if they were unable to provide valid self-reports due to severe health problems, such as delirium or severe dementia. All participants gave written informed consent and the study was approved by the local ethics committee of the University Hospital Halle (number 2022-026).

### 2.2 Variables of interest

The following variables of interest and covariates were selected based on the above-mentioned literature as well as to capture relevant clinical parameters such as cognition, functional ability and mood.

#### 2.2.1 Boredom proneness

Boredom proneness, was assessed using the Short Boredom Proneness Scale (SBPS), an eight-item self-report questionnaire. In our data, Cronbach’s Alpha was acceptable at 0.71. Participants rate each item on a five-point Likert scale, ranging from ‘never’ to ‘always’. Higher scores indicate a greater tendency to experience boredom. The scale captures typical indicators of boredom proneness, such as difficulty engaging in activities, lack of motivation, and feelings of monotony (Struk et al., 2017).

#### 2.2.2 Views on Ageing (VoA)

Domain-specific subjective VoA, which reflect individuals’ perceptions of ageing in different life domains was assessed using the “Individual Views on Ageing” questionnaire from the German Aging Survey (DEAS). This questionnaire captures views on age-related changes in four different domains: physical decline (reflecting the view that ageing is associated with physical losses, Cronbach *α* = 0.81), social losses (such as feeling less needed by others or experiencing less respect, Cronbach *α* = 0.64), continuous growth (implying that ageing is also seen as a time of continuing personal development, Cronbach *α* = 0.72), and self-knowledge (highlighting potential gains in self-perception, Cronbach *α* = 0.36). We calculated the sum of positive (continuous growth and self-knowledge) and negative VoA (social and physical losses), with higher values indicating more positive or negative VoA, respectively. Overall Cronbachs α for the total scale was 0.61, showing scores of *α* = 0.76 for the negative and *α* = 0.63 for the positive VoA scale. For subscale-specific analyses, we excluded the self-knowledge scale due to unacceptable Cronbachs *α* = 0.36. Participants rated each item on a scale from 1 (‘definitely true’) to 4 (‘definitely false’), with items reverse-scored for analysis (Dittmann-Kohli et al., 1997; Steverink et al., 2001; Wurm et al., 2007).

#### 2.2.3 Functional status

Functional status was measured using the Barthel Index (metric), which is performed by trained nurses as part of routine care upon admission and discharge from hospital. The Barthel Index describes level of function in ten activities of daily living such including eating, mobility, personal hygiene, and continence, with higher scores indicating independence (Mahoney and Barthel, 1965; Lübke et al., 2004). Especially in geriatric patients who suffer from acute clinical events, regaining independence in functional activities is a crucial therapy goal and reflects improvements in health status (Heimrich et al., 2025).

#### 2.2.4 Covariates

For all participants, we extracted sociodemographic and clinical parameters from the patient records to characterize the cohort:

- Age (years, metric), sex (male/female, dichotomous),
- Marital status (single/married/widowed/separated, multinominal),
- Living situation (with partner or family/alone or other, dichotomous),
- Education level (low, intermediate and high educational attainment, multinominal),
- Cognitive function measured by the Mini-Mental state examination (MMSE, metric) (Folstein et al., 1975),
- Geriatric depression scale (GDS, metric) assessing emotions and attitude to life with a maximum score of 15 points (Yesavage et al., 1982; Gauggel and Birkner, 1999; Krishnamoorthy et al., 2020). The GDS was specifically developed for use in older adults, with its good psychometric properties it is the most commonly used and recommended instrument to detect depressive symptoms in geriatric patients (Krishnamoorthy et al., 2020; Justo-Henriques et al., 2023).

### 2.3 Sample size calculation

A power analysis was conducted to determine the minimum sample size required for our primary multiple-regression analyses predicting rehabilitation gain (ΔBarthel) from baseline boredom proneness (SBPS) and covariates. Assuming a medium effect size (Cohen’s *f*^2^ = 0.15), an *α*-error probability of 0.05, and a desired power (1–*β*) of 0.80 with five predictors (SBPS, baseline Barthel, GDS, age, sex), the analysis indicated that *N* = 92 participants would be required. To achieve 90% power under the same conditions, the required sample increases to *N* = 108. Allowing for up to 10% attrition or missing data, we therefore targeted *N* = 120 inpatients, ensuring adequate statistical power for all planned regression and mixed-effects models.

### 2.4 Statistical analyses

All calculations were performed using SPSS 27.0 and R (version 4.1.3). The cohort was characterized using descriptive statistics [Mean (M), Standard Deviation (SD)]. The Shapiro–Wilk test was employed to test for normal distribution. Due to normality violations, group comparisons were tested using *U*-tests. The association between SBPS and other metric variables was tested using Spearman’s correlation. Linear multivariate regression analysis was carried out to characterize the association between SBPS and VoA with and without adjustment for covariates. Given the presence of heteroscedasticity, the regression analyses were adjusted using bootstrapping techniques (5,000 repeats) to ensure robust estimates. In order to account for the overlap between the VoA scale item 6 (“ageing means to me that I am bored more often”) and SBPS, we performed the regression analyses with and without item 6 included in the VoA sum score.

Lastly, to quantify the relationship between baseline boredom proneness, as measured by the SBPS, and functional changes during a two-week geriatric rehabilitation, we fit a longitudinal mixed-effects model with random intercepts for each patient, estimating fixed effects for time (admission vs. discharge), SBPS, and their interaction. All models were implemented in R using the lm and lmerTest packages, and model assumptions (linearity, normality of residuals, homoscedasticity) were confirmed via diagnostic plots. To confirm the mixed-effects model results, we applied two other complimentary analysis approaches as robustness checks. First, we regressed the change in Barthel Index (ΔBarthel) on SBPS alone and then sequentially adjusted for baseline Barthel, GDS, age, and sex using ordinary least squares. Second, we conducted an analysis of covariance (ANCOVA) predicting discharge Barthel scores from SBPS with baseline Barthel as a covariate. A significance level of *p* < 0.05 was considered significant.

## 3 Results

### 3.1 Description of the cohort

Table 1 summarizes the characteristics of the cohort. The mean age of participants was 83.4 years (SD = 6.4 years), with a predominance of females (70.8%) compared to males (29.2%). The majority were widowed or divorced (64.2%), with 65% living alone and 32.5% married. Educational attainment was low for most participants, with 66.7% reporting basic education, 17.5% having completed grade 10, and 15.8% having higher education (A levels or university degrees). Comprehensive geriatric assessments revealed that 25.8% had severe limitations based on the Barthel Index, and 73.3% reported moderate to severe difficulties with activities of daily living (Table 1).

**Table 1:** Baseline characteristics of the study population (*N* = 120).

Variable	Count (%) female	Count (%) male
Gender	85 (70.8)	35 (29.2)
	Mean (SD)	Range
Age, years	83.4 ± 6.39	62–101
Barthel index, admission	39.42 ± 13.92	10–100
Barthel index, discharge	50.93 ± 15.19	0–100
Mini mental state examination	27.09 ± 2.60	17–30
Geriatric depression scale	2.34 ± 2.13	0–9
Short Boredom Proneness Scale	13.08 ± 4.65	8–32
Negative Views on Ageing (sum)	20.53 ± 4.78	9–32
Positive Views on Ageing (sum)	22.55 ± 4.53	10–31
Views on Ageing physical losses	13.7 ± 2.98	5–16
Views on Ageing social losses	6.84 ± 2.80	4–16
Views on Ageing continuous growth	9.30 ± 3.21	4–16
Views on Ageing self-knowledge	13.2 ± 2.46	6–16

### 3.2 Views on Ageing

Participants’ VoA reflected both negative and positive beliefs. Regarding physical loss, the mean score was 13.69 (SD = 2.98), with 75% strongly agreeing that ageing reduces physical resilience and 64.2% acknowledging a decline in health. Social loss was associated with a mean score of 6.84 (SD = 2.81), indicating a perception of withdrawal. However, 60.8% disagreed with the inevitability of loneliness and 82.5% rejected the notion of diminished respect in older age. Positive VoA were highlighted in the areas of continuous growth (M = 9.30, SD = 3.21) and self-knowledge (M = 13.25, SD = 2.46) (see Table 1). About 40% believed they could continue to learn and grow, while other participants were less optimistic about improving skills (64.1%) or realizing ideas (58.4%). Notably, 75.8% agreed that ageing had improved their self-understanding and 74.2% reported increased relaxation over time.

### 3.3 Boredom proneness

**Figure 1:**
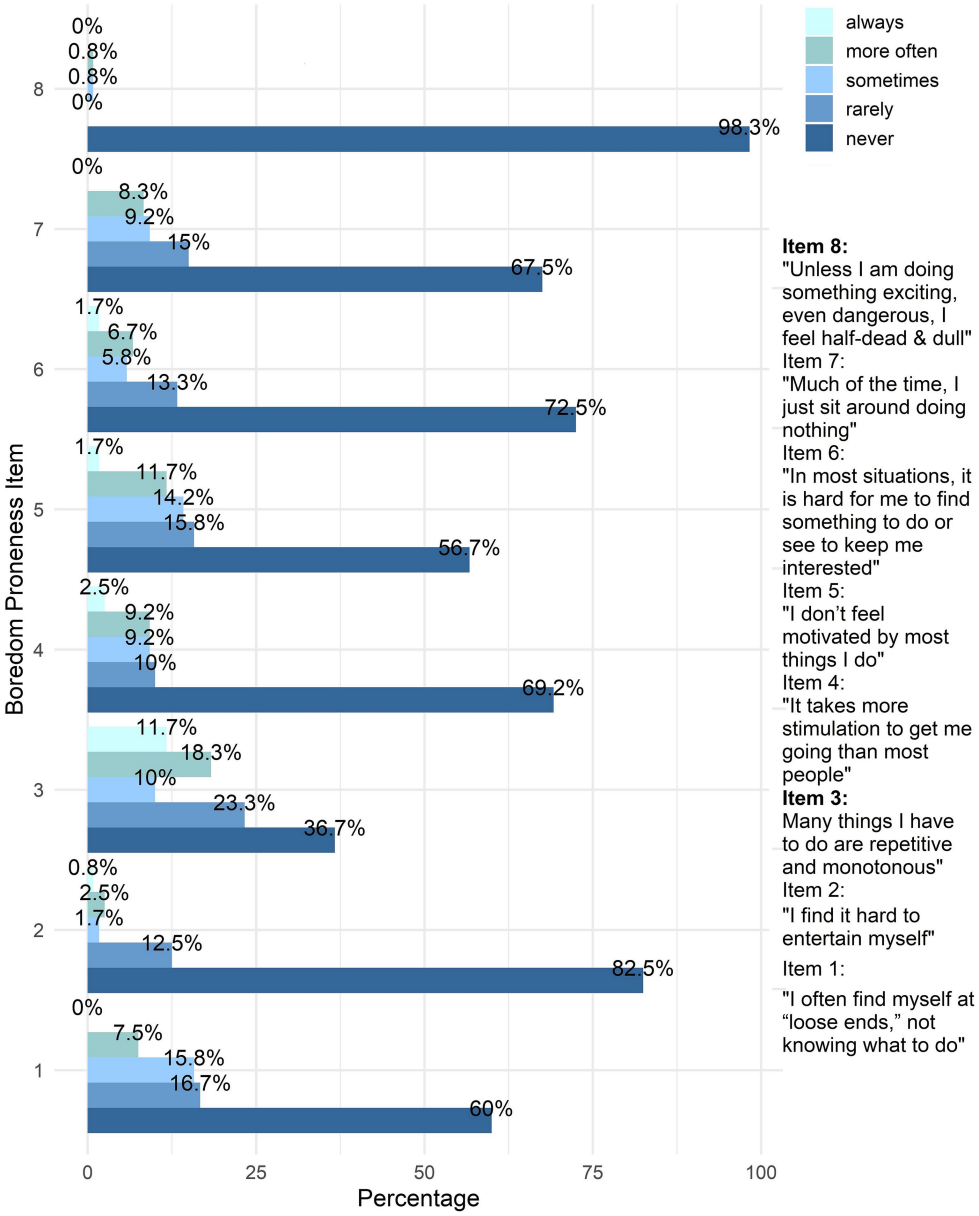
Response distribution across SBPS items. SBPS, Boredom Proneness Scale.

The descriptive analysis of the SBPS showed that the majority of older adults rarely experienced boredom proneness. 82.5% reported no difficulty in entertaining themselves and 98.3% did not feel “half dead and dull” unless engaged in something exciting (see Figure 1). However, certain aspects of boredom were more pronounced in a subset of participants. In particular, 30% more often or always found their activities repetitive or monotonous, while 36.7% never did. Similarly, 11.7% needed at least more often more stimulation to stay engaged and 13.4% felt unmotivated by many of their activities. Although most participants (67.5%) never felt idle, 17.5% sometimes or more often felt idle with nothing to do. On average, 10.4% of participants reported feeling bored at least more often.

**Figure 2:**
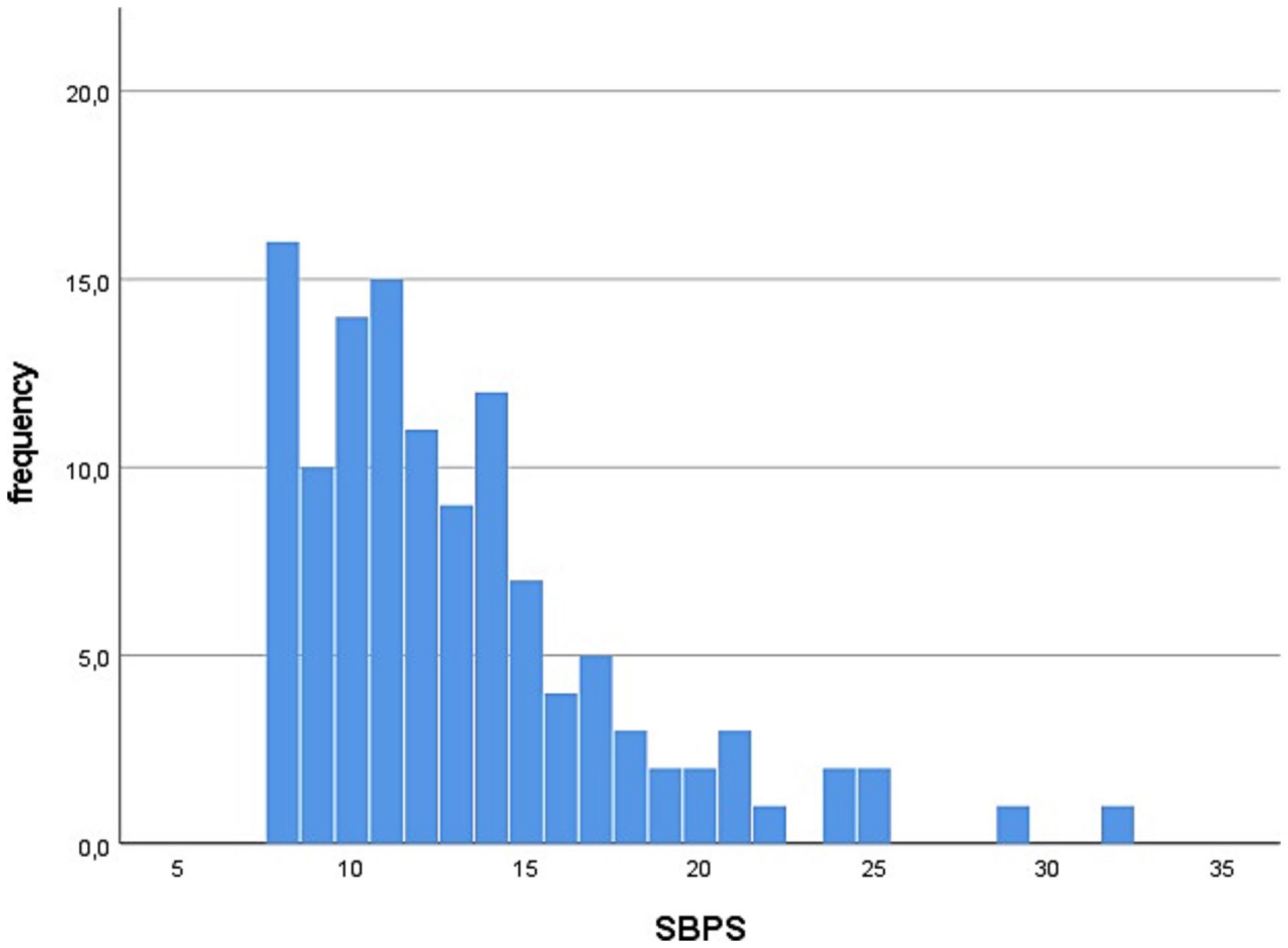
Distribution of boredom sum scores (SBPS).

Total SBPS scores were positively skewed, with most participants scoring at the lower end of the scale, indicating a low tendency to be bored (Figure 2). The mean score was 13.08 (SD = 4.65) (Table 1).

SBPS did not correlate with age (Supplementary Table 1). Women (mean = 12.0) had a higher SBPS compared to men (mean = 11.0; *p* = 0.029, *r* = −0.200) and participants living alone (mean = 12.5) reported higher SBPS than those living with a partner or family (mean = 11; *p* = 0.042, *r* = −0.186), although effect sizes were low.

### 3.4 Boredom proneness and VoA

Several linear regression models were used to study the association between boredom proneness and VoA in detail, with adjustment for covariates. In order to account for the overlap between the VoA scale item 6 (“ageing means to me that I am bored more often”) and SBPS, we performed the analyses with and without item 6 included in the VoA sum score.

Model 1 (Table 2) presents result including item 6 as well as the VoA subscales [*F*(9, 108) = 8.95, *p* < 0.001, adjusted R2 = 0.380]. The Durbin–Watson statistic was 1.92, indicating no strong violation of independence of residuals. Higher boredom proneness was significantly associated with age [*β* = − 0.17, 95% CI (−0.30, −0.05), *p* = 0.008], higher GDS score [*β* = 0.53, 95% CI (0.19, 0.88), *p* = 0.0031], as well as social losses [*β* = 0.67, 95% CI (0.39, 0.94), *p* < 0.001] and continuous growth [*β* = −0.26, 95% CI (−0.48, −0.03), *p* = 0.03], while physical losses did not have a significant effect (*p* = 0.237).

**Table 2:** Factors associated with boredom proneness according to linear regression.

Variable	Estimate	95% CI	*p*-value
Intercept	22.59	10.88–34.31	**<0.001**
Sex: male	−1.09	−2.74 – 0.55	0.190
Age	−0.17	−0.30 – −0.05	**0.008**
Living: not alone	0.21	−1.37 – 1.79	0.794
Educ [middle]	−0.39	−2.39 – 1.61	0.699
Educ [high]	−1.36	−3.21 – 0.50	0.149
GDS	0.53	0.19–0.88	**0.003**
VoAPhys	0.15	−0.10 – 0.39	0.237
VoASoc	0.67	0.39–0.94	**<0.001**
VoAGrow	−0.26	−0.48 – −0.03	**0.026**

*N* = 108, R2/adj. R2 = 0.427/0.380. *F*(9, 108) = 8.95.

VoA, Views of Ageing, Subscales Phys, physical losses, soc, social losses, grow, continuous growth. Note that the scale self-knowledge was not included in the analyses due to low internal consistency; GDS, Geriatric Depression Scale.

Bold values indicate statistical significance at *p* < 0.05.

This interpretation also translates into the results from the positive and negative VoA scales (Supplementary Table 2), with negative VoA [*β* = 0.39, 95% CI (0.25, 0.56), *p* < 0.001] associated with higher boredom but pot positive VoA [*β* = −0.13, 95% CI (−0.28, 0.02), *p* = 0.105]. For Model 2 with the exclusion of Item 6, no different results emerged, with comparison of the two models showing a highly similar pattern: the unstandardised effect of negative VoA on SBPS was essentially unchanged after removal of the single overlapping item [*B* = 0.393, 95% CI (0.249, 0.558) with item; *B* = 0.391, 95% CI (0.240, 0.575) without item]. Overall explained variance decreased marginally when the item was removed (Δ*R*^2^ = 0.023). These results indicate that the association between negative views on ageing and boredom proneness is robust and not solely attributable to item overlap.

### 3.5 Association between boredom proneness and functional outcome

We examined the extent to which baseline boredom proneness, as measured by the SBPS, predicts functional gains in Barthel Index during a two-week inpatient geriatric rehabilitation program.

**Figure 3:**
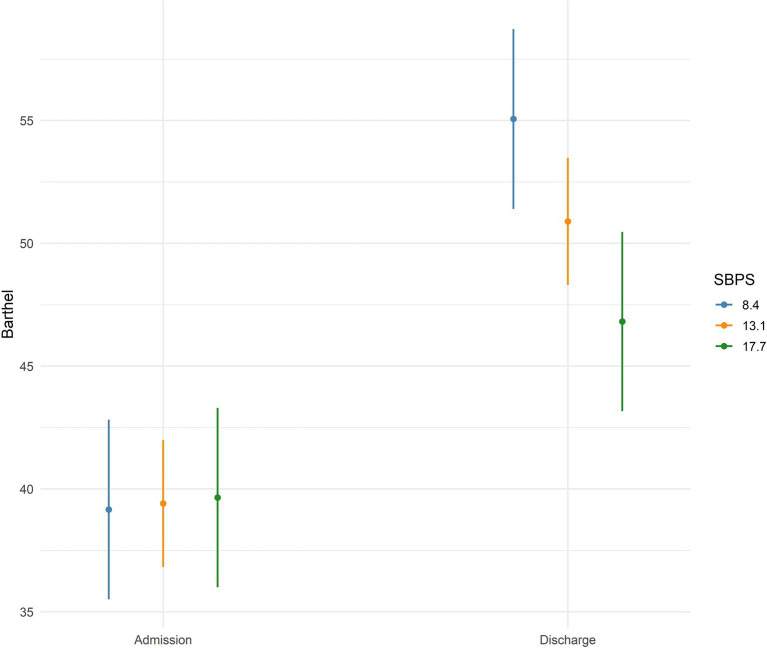
Effects of boredom proneness on change in Barthel index with time interaction. SBPS, Boredom Proneness Scale. The figure depicts the association between Barthel and boredom proneness at admission and discharge plotting the linear-mixed model results. The figure displays three levels of boredom proneness (M, ±1SD) to highlight the effect of strong boredom proneness (higher SBPS scores) on reduced Barthel improvement at discharge.

In the first step, a linear mixed-effects model (Table 3) using both admission and discharge Barthel scores revealed a robust Time × SBPS interaction (*β* = −0.94, SE = 0.36, *t* = −2.62, *p* = 0.010, Figure 3), indicating that for each one-point increase in SBPS, the average rehabilitation-related improvement in Barthel was reduced by 0.94 points. The main effect of Time was strong (*β* = 23.8, SE = 4.97, *t* = 4.78, *p* < 0.001), while the baseline SBPS-Barthel association at admission was nonsignificant (*β* = 0.05, SE = 0.28, *t* = 0.19, *p* = 0.853). The intra-class correlation for patient-level random intercepts was 0.188, indicating modest between-subject variability in overall functional trajectories, with marginal and conditional R2 set at 0.171 and 0.327. Together, these convergent analyses demonstrate that higher boredom proneness at admission reliably predicts diminished functional recovery in geriatric rehabilitation.

**Table 3:** Results of mixed-effects model for Barthel index improvement.

Variable	Est	*p*
Intercept	38.7	<0.001
Time	23.8	< 0.001
Baseline SBPS	0.05	0.853
Time * SBPS	−0.94	0.101

ICC (patient-random intercept): 0.188.

Marginal R2/Conditional R2: 0.171/0.327.

SBPS, Boredom Proneness Scale.

We computed the intra-class correlation (ICC) from the unconditional mixed model to quantify the proportion of total variance that is attributable to stable between-person differences. In our sample the ICC was 0.188, i.e., roughly 19% of total variance was between persons and ≈81% was within persons. This pattern is consistent with an inpatient rehabilitation setting in which functional status (Barthel) can change appreciably over the short treatment interval; consequently, within-person change and residual variation dominate the variance decomposition.

To confirm these results and ascertain the robustness of the results, we performed further analyses. In a series of linear regression models treating the change in Barthel Index (ΔBarthel) as the outcome (Supplementary Table 3), SBPS emerged as a significant negative predictor in the unadjusted model (*β* = −0.93, SE = 0.38, *t* = −2.48, *p* = 0.014; adj *R*^2^ = 0.043). When adjusting for baseline Barthel, the SBPS coefficient remained significant (*β* = −0.77, SE = 0.29, *t* = −2.67, *p* = 0.009), and the model explained substantially more variance (adj *R*^2^ = 0.431), reflecting both boredom proneness’ independent effect and the expected regression-to-the-mean of functional scores (Barthel admission *β* = −0.85, SE = 0.10, *t* = −8.88, *p* < 0.001). After additional adjustment for depressive symptoms (GDS), age, and sex did not attenuate the SBPS association (*β* = −0.84, SE = 0.33, *t* = −2.54, *p* = 0.012; adj *R*^2^ = 0.419), and GDS, age, and sex were nonsignificant (all *p* > 0.05). Although the VoA scales were insignificant in their effect on Barthel Index change (*p* > 0.005 for physical and social losses as well as continuous growth), their inclusion in the model (Supplementary Table 3 Model 4 and 5) slightly attenuated the effect of boredom proneness (*p* = 0.06) on Barthel Index change.

Lastly, complementing the change-score analyses, an ANCOVA predicting discharge Barthel with baseline Barthel and SBPS again showed a significant negative SBPS effect (*β* = −0.90, SE = 0.29, *t* = −3.14, *p* = 0.002; adj *R*^2^ = 0.092), confirming that higher boredom proneness at admission is associated with lower functional status at discharge independent of initial ability.

## 4 Discussion

In this study, we examined the association between VoA and boredom proneness in a sample of geriatric inpatients receiving early complex rehabilitation.

Negative VoA demonstrated robust associations with boredom proneness. As indicated in our regression analyses, these effects are primarily due to an effect of social losses more so than physical losses. These results support theoretical accounts proposing that internalization of negative age stereotypes fosters passivity, withdrawal, and a diminished sense of agency, all of which render individuals more susceptible to experiences of monotony and restlessness (von Humboldt et al., 2024). The subscale analyses suggest that this passivity and monotony may be due to social losses leaving participants unable to perform meaningful social activities. These results are in line with cumulative research citing social isolation and loneliness as more detrimental for well-being and quality of life than physical health, highlighting the importance of social connectedness in older age (Holt-Lunstad et al., 2015; Puvill et al., 2016; Courtin and Knapp, 2017). Clinically, patients who endorse beliefs of inevitable decline may be less inclined to engage in stimulating or goal-directed activities, thereby exacerbating proneness for boredom, as indicated by the negative association between the VoA subscale continuous growth and boredom proneness in our data. This interpretation is also in line with the close link between boredom proneness and feelings of control, which are diminished in persons with negative VoA who believe that they cannot influence their aging process (Wolff and Martarelli, 2020). Likewise, a recent study on flow experiences, an “antagonist” of boredom reflecting full submersion in a task and representing a sense of control over said task, was linked to positive affect and reduced negative VoA (Finnigan et al., 2025). Philosophical analyses highlight that loss of continuity in life projects can undermine practical identity and foster boredom. Our data suggest two non-exclusive mechanisms: negative VoA may reflect an expectation of disrupted continuity, and they may reduce agency and exploration needed to form new roles. Clinically, interventions can either preserve continuity or actively support adaptive re-engagement (goal-setting, skills training).(Weinstein et al., 1995; Fahlman et al., 2009). For example, in their study of retirees, Weinstein and colleagues found a significant negative correlation between subjective life purpose and boredom. Additionally, retirees who volunteered more than ten hours per week reported significantly lower boredom scores compared to those who volunteered less (Weinstein et al., 1995). Further evidence suggests that older adults who view aging as an opportunity for self-reflection, continuous growth, and meaning-making are less vulnerable to boredom. These individuals are more likely to engage in novel learning experiences and creative pursuits, which stimulate cognitive functioning and foster intrinsic motivation (Freund and Baltes, 1998). Mechanistically, a growth mindset may motivate older adults to pursue novel learning opportunities and meaningful social roles, thereby mitigating situational monotony. In line with the idea that state boredom signals the need for redirection of attention and changing of situational circumstances, the absence of negative VoA such as social losses may enable older adults to seek out novel situations while present negative VoA signal the disbelief in such a continuous growth (Bieleke et al., 2022). Likewise, VoA may serve as self-fulfilling prophecies: applying the Stereotype Embodiment Theory on aging (Levy, 2009) suggests that activating negative VoA subconsciously leads to concordant behavior that affirms these VoA. In case of a health event such as hospitalization, negative VoA may lead to patients employing less favourable coping strategies (Wurm et al., 2013) and instead result in less activating and engaging behavior, thus increasing boredom. These results indicate that expectations towards aging influence cognitive appraisal of situations as well as behavior. Rehabilitation programs that incorporate control perceptions, goal-setting, skill development, and opportunities for mastery may therefore reinforce growth-oriented VoA and reduce boredom.

Further analyses examined how boredom proneness upon admission related to functional gains during early rehabilitative geriatric treatment, as measured by changes in the Barthel Index. Using different methods, we were able to show that individuals more prone to boredom may derive less functional benefit from rehabilitation. Several hypotheses may account for this link, and each warrants empirical investigation in future trials. One possibility is that boredom proneness reflects a broader deficit in intrinsic motivation and goal-directed engagement, such that patients who report greater boredom proneness may devote less effort to physiotherapy, occupational therapy, and self-care activities (Wolff et al., 2021). A related hypothesis is that proneness to boredom signals low self-efficacy or self-control (Wolff and Martarelli, 2020), which could diminish patients’ confidence in their capacity to improve and thereby undermine adherence to prescribed exercises. From a cognitive-behavioral standpoint, it is plausible that boredom proneness is associated with impairments in attentional control and executive function, making it more difficult to sustain focus on complex rehabilitation tasks. Finally, boredom proneness may proxy for inadequate social stimulation or meaningful interpersonal interaction during the rehabilitation stay, a factor that could erode the motivational support typically provided by peers and caregivers. Each of these conjectured mechanisms—motivational, cognitive, and social—should be formally tested in longitudinal and interventional studies to determine whether targeting boredom proneness directly can enhance rehabilitative engagement and improve functional outcomes.

In addition, depressive symptoms independently predicted higher boredom proneness, confirming earlier findings that affective distress constitutes a central antecedent of boredom (Watt and Vodanovich, 1999; Isacescu et al., 2017). Goldberg et al. (2011) and Malkovsky et al. (2012) reported a significant correlation between depressive symptoms and boredom in both student (Goldberg et al., 2011) and clinical populations (Malkovsky et al., 2012). From the perspective of depression’s hallmark features—anhedonia, loss of interest, and diminished positive affect—boredom, conceptualized as a lack of meaning and goal-directed engagement, emerges not merely as a comorbid phenomenon but as an integral manifestation of the underlying disruption in affective processing (Goldberg et al., 2011; Isacescu et al., 2017; Van Tilburg and Igou, 2017). Accordingly, interventions should address not only maladaptive ageing beliefs but also the identification and treatment of depressive symptomatology in order to alleviate the risk of boredom and its downstream consequences.

While this study provides an insight into the previously underexplored topic of boredom proneness in geriatric patients, it is subject to several limitations. First, the cross-sectional design precludes causal inference regarding the directionality of observed associations between boredom and VoA; longitudinal research is required to determine whether negative VoA precipitate increased boredom or whether chronic boredom fosters more negative ageing perceptions. This longitudinal design should further include longer follow-up spans to assess long-term changes in functional health and their relation with boredom. In this context, we computed the intra-class correlation (ICC) from the unconditional mixed model on Barthel change to quantify the proportion of total variance that is attributable to stable between-person differences. In our sample the ICC was 0.188, i.e., roughly 19% of total variance was between persons and ≈81% was within persons. This pattern is consistent with an inpatient rehabilitation setting in which functional status (Barthel) can change appreciably over the short treatment interval; consequently, within-person change and residual variation dominate the variance decomposition. In the present study, we assessed the effect of VoA on boredom in order to understand boredom’s underexplored influence on health; however, the reverse association may also be present in a bi-directional link. In future studies, to fully understand the association between VoA and boredom proneness, the direction of effects should be further explored, especially under consideration of additional covariates such as chronic pain, social network size, and activity level. Especially self-efficacy and control perception should be incorporated in the analyses to assess their robustness and uncover potential pathways linking VoA and boredom. Our reliance on self-report measures may introduce social desirability bias, however, as boredom is defined as a subjective experience, objective measures are not feasible. Likewise, both VoA and boredom were measured with only one of many available instruments and their measurements should be repeated with different tools in future studies. This is especially important as the included patients showed low levels of boredom, leading to a potential underestimation of the effects which should be confirmed in patients with a wider range of boredom scores. Additionally, the inclusion of biomarkers of aging may enhance the understanding of the interplay between psychosocial and biological processes in future studies. In future studies, the addition of molecular biomarkers of aging, which were not available in the present dataset, may introduce more clarity on the biological processes and effects of boredom and VoA. Likewise, the applicability of the findings to broader populations may be limited by regional and sample-specific factors. While the inpatient rehabilitation sample of older patients limits generalizability to community-dwelling older adults, the impact of boredom may be particularly pronounced in this population due to health restrictions that increase sedentary behavior and reduce the participation in activities, making boredom a highly relevant topic for these patients. In light of the cross-sectional design, we aimed to increase the robustness of our analyses using the following steps: covariate adjustment for key health indicators (Barthel, Tinetti, MMSE), robustness analyses (VoA scale with/without the overlapping boredom item), bootstrap inference for coefficient stability, and triangulation across multiple analytic models (change-score, ANCOVA, mixed models).

Nevertheless, this study advances the literature by identifying VoA as modifiable cognitive factors associated with boredom proneness in geriatric populations. From a clinical perspective, routine assessment of VoA and boredom alongside mood screening may help to identify patients at elevated risk for disengagement. Interventions such as cognitive restructuring, life review therapy, and strength-based group sessions could be employed to challenge maladaptive ageing beliefs and foster growth-oriented perspectives. In addition, programs designed to compensate for perceived social losses—for example, structured peer-support groups or community engagement initiatives—may address both cognitive and social risk factors for boredom.

### 4.1 Conclusion

In our study on geriatric patients, boredom proneness as a state variable has been linked with worse outcomes in functional health for geriatric patients. Boredom proneness in this population is associated with higher depressive symptoms and more negative VoA, and previous research suggests maladaptive health behavior due to lack of engagement and control beliefs as a potential mechanism for these effects. Future research should employ longitudinal, biomedical and experimental designs to understand the link between boredom proneness and health outcomes and to evaluate the efficacy of VoA-focused interventions on reducing boredom proneness and improving rehabilitative outcomes. Studies conducted in diverse cultural and care settings are needed to establish the generalizability of these findings. Moreover, qualitative investigations into older adults’ subjective experiences of ageing and boredom could provide nuanced insights to refine intervention content.

## Data Availability

The raw data supporting the conclusions of this article will be made available by the authors, without undue reservation.
