# Supplementary material for: The association between boredom proneness, functional status, and views on ageing in geriatric patients
Source: Front Psychol. 2025 Nov 6;16:1657437. doi: 10.3389/fpsyg.2025.1657437 (PMC12631226; doi:10.3389/fpsyg.2025.1657437)
Supplement: Supplementary file 1 [file Table_1.DOCX]

Supplementary Materials to **The Association between Boredom Proneness, Functional Status, and Views on Ageing and Boredom in Geriatric Patients**

**Supplement Table 1.** Associations between boredom proneness (SBPS) and covariates

| **Variable** | **Spearman r** | **p-value** |
| --- | --- | --- |
| Age | -.1 | .29 |
| GDS | .38 | < .001 |
| Barthel admission | -.04 | .68 |
| VOA Negative | .49 | < .001 |
| VOA Positive | -.15 | .10 |
| MMSE | -.09 | .34 |
|  | **Wilcoxon Test r** | **p-value** |
| Sex | .20 | .03 |
| Marital | .22 | .02 |
| Living | .19 | .04 |
|  | **Kruskal Wallis Test** | **p-value** |
| Education | .04 | .05 |
| GDS = Geriatric Depression Scale, MMSE =Mini Mental State Examination, VoA = Views of Ageing | | |

**Supplement Table 2.** Linear Regression for boredom proneness and views of ageing subscales

| ***Predictors*** | ***Estimates*** | ***CI*** | ***p*** |
| --- | --- | --- | --- |
| (Intercept) | 21.53 | 9.05, 35.07 | < .001 |
| Sex [male] | -1.01 | −2.60, 0.71 | .061 |
| Age | -0.16 | −0.31, −0.04 | < .001 |
| LivingSit [alone] | 0.45 | −1.20, 1.98 | .581 |
| Educ | -0.86 | −1.66, −0.02 | .061 |
| GDS | 0.62 | 0.19, 1.02 | < .001 |
| Positive VoA | 0.39 | 0.25, 0.56 | < .001 |
| Negative VoA | -0.13 | −0.28, 0.02 | .105 |
| (F(7,110) = 9.84, p < 0.001). R² = 0.385; adjusted R² = 0.346  GDS = geriatric depression scale, VoA = views of ageing | | | |

**Supplement Table 3**. Ordinary Least-Squares Regression on Barthel Index Change

| **Model 1**, adj. R2 = .043 | **Est** | ***p*** |
| --- | --- | --- |
| Intercept | 28.3 | <.001 |
| SBPS | -0.93 | .014 |
| **Model 2**, R2 = .431 | **Est** | ***p*** |
| Intercept | 55.4 | <.001 |
| SBPS | -0.77 | .009 |
| Barthel admission | -0.85 | <.001 |
| **Model 3,** R2 = .419 | **Est** | ***p*** |
| Intercept | 62.7 | .001 |
| SBPS | -0.84 | .012 |
| Barthel admission | -0.85 | <.001 |
| GDS | 0.36 | .613 |
| Age | 0.09 | .684 |
| Sex male | 1.11 | .719 |
| **Model 4, R2 = 0.412** | **Est** | ***p*** |
| Intercept | 56.8 | 0.17 |
| SBPS | -0.73 | 0.06 |
| Barthel admission | -0.849 | < .001 |
| GDS | 0.426 | 0.558 |
| Age | -0.004 | .989 |
| Sex male | 1.13 | .716 |
| VoA Physical Losses | -0.513 | 0.315 |
| VoA Social Losses | 0.151 | 0.806 |
| VoA Continuous Growth | 0.341 | 0.463 |
| Note: SBPS = boredom proneness scale, GDS = Geriatric Depression Scale, VoA = Views on Ageing | | |
